# Pretransplant and perioperative predictors of early heart transplantation outcomes

**DOI:** 10.3325/cmj.2014.55.553

**Published:** 2014-12

**Authors:** Hrvoje Gašparović, Stjepan Ivanković, Jana Ljubas Maček, Filip Matovinović, Mislav Nedić, Lucija Svetina, Maja Čikes, Boško Skorić, Željko Baričević, Višnja Ivančan, Bojan Biočina, Davor Miličić

**Affiliations:** 1 Department of Cardiac Surgery, Clinical Hospital Center Zagreb, University of Zagreb, Zagreb, Croatia; 2 Department of Cardiac Surgery, Clinical Hospital Center Zagreb, University of Zagreb, Zagreb, Croatia

## Abstract

**Aim:**

To identify predictors of 3-month mortality after heart transplantation in a Croatian academic center.

**Methods:**

A retrospective review of institutional database identified 117 heart transplantations from January 2008 to July 2014. Two children <14 years were excluded from the study. The remaining 115 patients were dichotomized into survivors and non-survivors adjudicated at 3-months postoperatively, and their demographic, clinical, and longitudinal hemodynamic data were analyzed.

**Results:**

3-month survival after heart transplantation was 86%. Non-survivors were older (59 ± 8 vs 50 ± 14 years, *P* = 0.009), more likely to have previous cardiac surgery (44% vs 19%; odds ratio [OR] 3.28, 95% confidence interval [CI] 1.08-9.90; *P* = 0.029), lower body mass index (BMI) (25 ± 4 vs 28 ± 2 kg/m^2^, *P* = 0.001), and be diabetics (44% vs 23%; OR 2.57, 95% CI 0.86-7.66; *P* = 0.083). Creatinine clearance was marginally superior among survivors (59 ± 19 vs 48 ± 20 mL/min, *P* = 0.059). Donor age and sex did not affect outcomes. Non-survivors were more likely to have had ischemic cardiomyopathy (69% vs 32%, *P* = 0.010). Postoperative utilization of epinephrine as a second line inotropic agent was a strong predictor of mortality (63% vs 7%; OR 21.91; 95% CI 6.15-78.06; *P* < 0.001). Serum lactate concentrations were consistently higher among non-survivors, with the difference being most pronounced 2 hours after cardiopulmonary bypass (9.8 ± 3.5 vs 5.2 ± 3.2 mmol/L, *P* < 0.001). The donor hearts exhibited inferior early hemodynamics in non-survivors (cardiac index 3.0 ± 1.0 vs 4.0 ± 1.1 L/min/m^2^, *P* = 0.001), stroke volume (49 ± 24 vs 59 ± 19 mL, *P* = 0.063), and left and right ventricular stroke work indices (18 ± 8 vs 30 ± 11 g/beat/m^2^, *P* < 0.001 and 5 ± 3 vs 7 ± 4 g/beat/m^2^, *P* = 0.060, respectively). Non-survivors were more likely to require postoperative re-sternotomy (50% vs 12%; OR 7.25, 95% CI 2.29-22.92; *P* < 0.001), renal replacement therapy (RRT) (69% vs 9%; OR 22.00, 95% CI 6.24-77.54; *P* < 0.001), and mechanical circulatory assistance (MCS) (44% vs 5%; OR 14.62, 95% CI 3.84-55.62; *P* < 0.001). Binary logistic regression revealed recipient age (*P* = 0.024), serum lactates 2 hours after CPB (*P* = 0.007), and epinephrine use on postoperative day 1 (*P* = 0.007) to be independently associated with 3-month mortality.

**Conclusion:**

Pretransplant predictors of adverse outcome after heart transplantation were recipient age, lower BMI, ischemic cardiomyopathy, reoperation and diabetes. Postoperative predictors of mortality were inferior donor heart hemodynamics, epinephrine use, and serum lactate concentrations. Non-survivors were more likely to require re-sternotomy, MCS, and RRT.

Heart failure (HF) presents a major public health burden, and its management consumes a large proportion of the health care budget (1,2). While the clinical syndrome of HF is multifactorial in origin, its cardinal symptoms are remarkably similar irrespective of the diverse underlying cardiac pathology. The adverse impact of HF on the quality of life and overall mortality has brought the issue into focus of the contemporary medical community. The already high financial burden of HF will likely increase in parallel to increasing age of the general population. Contrariwise, the number of orthotopic heart transplantation (OHT) worldwide has plateaued over the past decade (3). The unmatched need for donor organs has served as a strong impetus for the development of alternative lines of management, including mechanical circulatory assistance. While we have witnessed both an accelerated evolution of ventricular assist devices and their wider dissemination within the HF population, the high incidences of associated complications reduce the effectiveness of this line of management. The contemporary armamentarium for HF management also includes pharmaceutical modulation of multiple targets and biventricular pacing. This complex array of management tools notwithstanding, OHT remains the unchallenged gold standard when it comes to long-term outcomes for patients with end-stage HF (4). The importance of effective utilization of available organs is paramount in the setting of a pronounced shortage of suitable allografts. The aim of this study was to identify the predictors of adverse 3-month outcome following heart transplantation in a tertiary-care academic center.

## Methods

### Study design

We conducted a retrospective chart review of all heart transplant recipients aged over 14 years, operated on at the University Hospital Center Zagreb, Croatia from January 1, 2008 to July 15, 2014. The local Institutional Review Board approved the study. Written informed consent was waived due to the retrospective nature of the study.

### Data collection, variables examined, and outcome measure

During the observed study period 117 heart transplantations were performed and were screened for study enrollment. Of these, 2 were performed in children aged <14 years. These were excluded from further analysis. After identifying cases from the institutional electronic database, individual medical records were reviewed for demographic, clinical, history, laboratory and hemodynamic data. Studied donor variables were donor age and sex. Comorbidities examined were diabetes, hypertension, hyperlipidemia, smoking, and atrial fibrillation. Pulmonary artery catheters (Argon Medical Devices, Singapore) were placed universally through the jugular vein. Their correct positioning was based on pressure tracings. Thermodilution hemodynamic data were obtained in triplicate and then averaged. Hemodynamic measurements included cardiac index, stroke volume, stroke work indices for both ventricles, central venous and pulmonary capillary wedge pressures, as well as systemic and pulmonary vascular resistances. Blood samples and hemodynamic data acquired 2 hours after CPB and on postoperative days 1 and 7 were used. The primary outcome of the study was 3-month mortality.

### Allograft implantation procedure

Every effort was made to reduce the allograft ischemic time by frequent communications between the procurement and implanting teams. Ideally, a donor ischemic time of less than 4 hours was aimed for. The University of Wisconsin solution was used for myocardial protection and cold storage from 2008 to 2012. Custodiol solution was used for the same purpose during the latter two years of the study period. The preferred allograft implantation approach was the bicaval technique during tepid cardiopulmonary bypass (CPB). The left atrial and aortic anastomoses were performed on a cross-clamped aorta. The remaining anastomoses were performed on CPB with the heart perfused. The donor heart rate, after weaning from CPB, was maintained between 100-120 beats per minute by a combination of positive chronotropic agents and atrial pacing. Isoproterenol was our preferred agent for this purpose. Inotropic support typically included dobutamine or milrinone, or a combination of these agents. Epinephrine was used only when further escalation of inotropic support was deemed warranted. Nitric oxide was not used routinely. Synthetic prostaglandin E1 was used selectively in patients with pulmonary hypertension or elevated pulmonary vascular resistance. Norepinephrine was used to counteract low systemic vascular resistance. Any evidence of hypoperfusion would have prompted escalation of inotropic support. In the event that pharmacological cardiac support alone was insufficient, a low threshold for instituting mechanical circulatory assistance (MCS) was practiced. The first line MCS for this indication was extracorporeal membrane oxygenation. Right ventricular assist devices were used in select cases.

### Immunosuppressive protocol

We relied on antithymocyte globulin for the induction of immunosuppression. Our institutional protocol included a combination of cyclosporine A, mycophenolate mofetil, and steroids for maintenance of immunosuppression. In select cases tacrolimus was used instead of cyclosporine A. An attempt was made to wean steroids off, beginning no sooner than six months after OHT. Individual immunosuppression drug dosages were tailored based on trough levels. Right ventricular endomyocardial biopsies were performed every 7-14 days over the early postoperative period. Rejection was graded in line with the revised International Society for Heart and Lung Transplantation classification (5).

### Statistical analysis

The continuous data are presented as mean values with their standard deviations, or absolute numbers and percentages. Categorical variables are presented as absolute numbers and percentages. Missing data were addressed using multiple imputations based on the Markov-chain Monte Carlo procedure to generate 20 imputed data sets during 2500 iterations. The percentages of missing data for the studied variables varied between 0% and 26%. Over 50% of the studied variables had less than 10% of missing data. Over 90% of studied variables had less than 25% of missing data. The number of imputation used in the study provided a relative efficiency of 0.987. Normality of distribution was tested with the Shapiro-Wilk test. Mann-Whitney U test was utilized for data with non-normal distribution, whereas *t* test was used for analyzing continuous data following a normal distribution.

Variables found to be significantly associated with adverse outcome on univariate analysis were included in a binary logistic regression model. These included serum lactate concentrations and hemodynamic data found to be associated with adverse outcomes on univariate analysis. Age and body mass index (BMI) completed the list of covariates fit into the regression model. Categorical variables were compared with the χ^2^ test. Odds ratios were used as a measure of the association between specific comorbidities/clinical characteristics and 3-month mortality. The respective 95% confidence intervals (CI) were provided. A two-tailed *P* value <0.05 was considered to be statistically significant. The data were processed using the IBM SPSS Statistics software package (version 22.0; Somers, NY, USA).

## Results

### Baseline patient characteristics

Of the total of 117 patients who underwent ortothopic heart transplantation at our center, 115 met the inclusion criteria and were included in this analysis. There was no difference in sex between patients who survived 3 months after OHT and those who did not (Table 1). Non-survivors were older (59 ± 8 vs 50 ± 14 years; *P* = 0.009). The incidence of diabetes mellitus was marginally higher among non-survivors (44% vs 23%; OR 2.57, 95% CI 0.86-7.66; *P* = 0.083). The impact of lower creatinine clearance on 3-month mortality was just below the predefined threshold of statistical significance (non-survivors: 48 ± 20 vs survivors: 59 ± 19 mL/min; *P* = 0.059) (Table 2). Pulmonary vascular resistances (PVR) and N-terminal prohormone of brain natriuretic peptide values were not different between the groups (Table 2). The primary cardiac pathology among survivors was dilatative cardiomyopathy (58% vs 31%; *P* = 0.061) (Table 3). Conversely, ischemic cardiomyopathy was more prevalent among non-survivors (69% vs 32%; *P* = 0.010). Of note, the preoperative use of MCS did not adversely affect the outcome (Table 1).

**Table 1:** Patient demographic and clinical data*

	3-month outcome			
Preoperative descriptors	non-survivors, n (%)	survivors, n (%)	Odds ratio (95% CI)	*P*
Male recipients	14 (88)	72 (73)	2.63 (0.56-12.32)	0.207
Male donors	11 (69)	69 (70)	0.957 (0.306-2.993)	0.939
Hyperlipidemia	4 (25)	31 (31)	0.73 (0.22-2.45)	0.611
Hypertension	7 (44)	34 (34)	1.49 (0.51-4.34)	0.466
Diabetes mellitus	7 (44)	23 (23)	2.57 (0.86-7.66)	0.083
Smoking history	3 (19)	10 (10)	2.05 (0.50-8.46)	0.311
Atrial fibrillation	8 (50)	30 (30)	2.30 (0.79-6.70)	0.120
Reoperation	7 (44)	19 (19)	3.28 (1.08-9.90)	0.029
Preoperative inotropic support (any)	6 (38)	36 (36)	1.05 (0.35-3.13)	0.930
Preoperative epinephrine	3 (19)	7 (7)	3.03 (0.70-13.22)	0.124
Preoperative MCS	5 (31)	17 (17)	2.19 (0.67-7.13)	0.184

*Abbreviations: CI – confidence intervals; MCS – mechanical circulatory assistance.

**Table 2:** Continuous descriptors of patient cohorts*

	3-month outcome		
	non-survivors, mean (SD)	survivors, mean (SD)	*P*
**Preoperative descriptors**			
Recipient age (years)	59 (8)	50 (14)	0.009
Donor age (years)	38 (13)	38 (12)	0.916
Body mass index (kg/m^2^)	25 (4)	28 (2)	0.001
Creatinine clearance (mL/min)	48 (20)	59 (19)	0.059
NT-proBNP	7559 (4380)	8009 (6738)	0.701
Preoperative lactate (mmol/L)	2.8 (1.4)	2.6 (2.4)	0.167
PVR	242 (122)	247 (137)	0.890
**Peri-procedural descriptors**			
Ischemic time	193 (58)	178 (61)	0.355
CPB duration (min)	194 (70)	170 (72)	0.145
Mechanical ventilation (hrs)	266 (177)	46 (52)	<0.001

*Abbreviations: SD – standard deviation; NT-proBNP – N-terminal prohormone of brain natriuretic peptide; PVR – pulmonary vascular resistance; CPB – cardiopulmonary bypass.

**Table 3:** Primary cardiac pathology antedating heart transplantation*

	3-month outcome		
	non-survivors, n (%)	survivors, n (%)	*P*
Dilatative CMP	5 (31)	57 (58)	0.061
Ischemic CMP	11 (69)	32 (32)	0.010
Valvular CMP	0 (0)	2 (2)	1.0
Restrictive CMP	0 (0)	2 (2)	1.0
Hypertrophic obstructive CMP	0 (0)	2 (2)	1.0
Grown-up congenital disease	0 (0)	2 (2)	1.0
ARVD	0 (0)	2 (2)	1.0

*Abbreviations: CMP – cardiomyopathy; ARVD – arrhythmogenic right ventricular dysplasia.

### 3-month outcome

**Figure 1:**
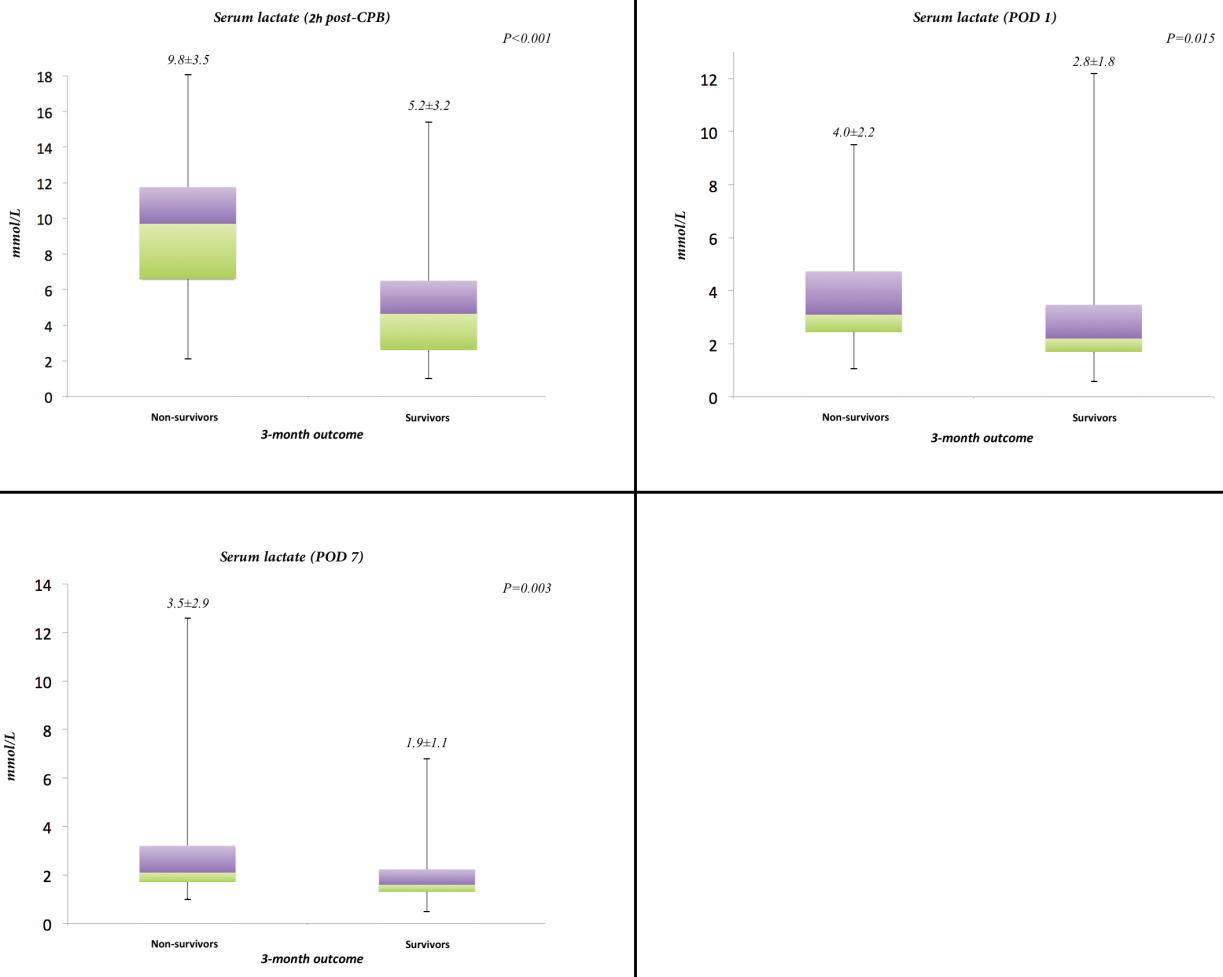
Box and whisker plots showing the serum lactate concentrations in survivors and non-survivors over the early postoperative period. Boxes represent the 25th and 75th percentiles, and horizontal lines within the boxes are the median values. The whiskers illustrate the minimum and maximum values.

Three-month survival after OHT was 86% (99/115). The causes of death were sepsis (9 patients), RV failure (3 patients), stroke (1 patient), mesenteric ischemia (1 patient), and bleeding (2 patients). Non-survivors were never discharged from the hospital. The donor ischemic times were similar between survivors and non-survivors (Table 2). A quarter of non-survivors and 17% of survivors had ischemic times that exceeded 4 hours (*P* = 0.452). Neither donor sex nor donor age was predictive of 3-month mortality (Tables 1 and 2). The prevalence of donors older than 50 years was similar between non-survivors and survivors (19% vs 17%; *P* = 0.877). Among the most robust predictors of adverse 3-month outcome were the serum lactate concentrations. Non-survivors had higher serum lactate concentrations over the early postoperative course (Figure 1). The predictive value of serum lactates on 3-month survival was highest for the values taken 2 hours after CPB (non-survivors: 9.8 ± 3.5 vs survivors: 5.2*±*3.2 mmol/L; *P* < 0.001). Postoperative inotropic support was employed almost universally in the immediate post-procedural period, without any difference between survivors and non-survivors. However, the use of epinephrine as an inotropic agent was linked to 3-month mortality (Table 4). Epinephrine use was limited to patients requiring a second line inotropic agent. Patients who required epinephrine on postoperative day 1 were significantly more likely to die within 3 months of OHT (63% vs 7%; OR 21.91, 95% CI 6.15-78.06; *P* < 0.001). A similar trend was seen in patients in whom epinephrine was administered both immediately after CPB and as far into their postoperative course as day 7 post-OHT (Table 4). Non-survivors were more likely to require at least one re-sternotomy for any reason (50% vs 12%; OR 7.25, 95% CI 2.29-22.92; *P* < 0.001). Postoperative MCS was employed in patients in whom pharmacological augmentation of cardiac function proved insufficient for maintaining optimal systemic perfusion. Clearly, this scenario was much more common among non-survivors (44% vs 5%; OR 14.62, 95% CI 3.84-55.62; *P* < 0.001). Non-survivors also required longer periods of mechanical ventilation (266 ± 177 vs 46 ± 52 hours; *P* < 0.001).

**Table 4:** Postprocedural patient characteristics*

	3-month outcome			
	non-survivors, n (%)	survivors, n (%)	Odds ratio (95% CI)	*P*
**Postoperative cardiac support**				
Any inotrope T1	15 (94)	91 (92)	1.32 (0.15-11.31)	0.800
Epinephrine T1	6 (38)	11 (11)	4.80 (1.46-15.79)	0.006
Any inotrope T2	16 (100)	84 (85)	1.19 (1.09-1.30)^†^	0.095
Epinephrine T2	10 (63)	7 (7)	21.91 (6.15-78.06)	<0.001
Any inotrope T3	11 (85)	62 (63)	3.28 (0.69-15.63)	0.118
Epinephrine T3	7 (54)	19 (19)	4.91 (1.48-16.30)	0.005
Postoperative MCS	7 (44)	5 (5)	14.62 (3.84-55.62)	<0.001
**Postoperative complications**				
RRT	11 (69)	9 (9)	22.00 (6.24-77.54)	<0.001
CVI	2 (13)	4 (4)	3.39 (0.57-20.28)	0.158
Re-sternotomy	8 (50)	12 (12)	7.25 (2.29-22.92)	<0.001

*Abbreviations: CI – confidence intervals; T1 – 2 hours post CPB; T2 – postoperative day 1; T3 – postoperative day 7; MCS – mechanical circulatory support; RRT – renal replacement therapy; CVI – cerebrovascular incident.

†Relative risk.

### Hemodynamic data

**Figure 2:**
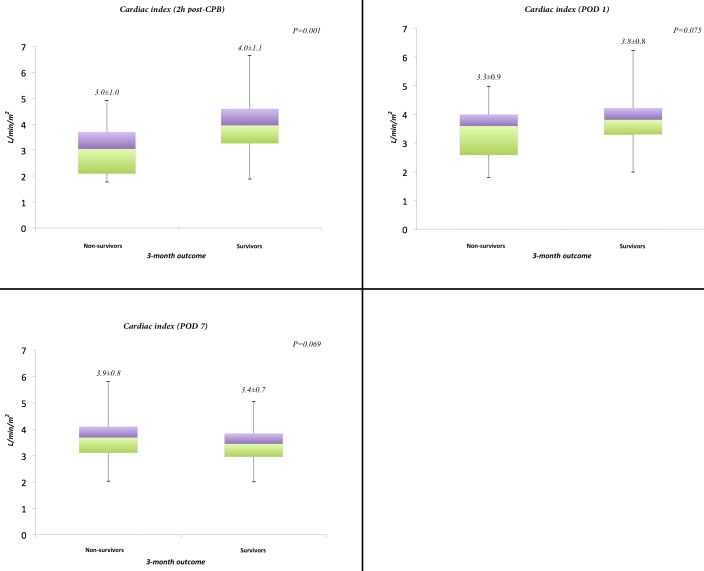
Box and whisker plots depicting the thermodilution-derived cardiac index in survivors and non-survivors over the early postoperative period. Boxes represent the 25th and 75th percentiles, and horizontal lines within the boxes are the median values. The whiskers illustrate the minimum and maximum values.

Preoperative hemodynamics of the recipient’s native hearts did not affect 3-month postoperative outcome (Table 5). Contrariwise, the hemodynamic performance of the transplanted donor heart had a significant effect on 3-month mortality (Table 5, Figure 2). Cardiac index (CI) and stroke volume (SV) 2 hours after CPB were lower in non-survivors (3.0 ± 1.0 vs 4.0 ± 1.1 L/min/m^2^; *P* = 0.001 and 49 ± 24 vs 59 ± 19 mL; *P* = 0.063, respectively). Left and right ventricular stroke work indices calculated simultaneously with the aforementioned hemodynamic parameters were also lower in non-survivors (left ventricular stroke work index [LVSWI]: 18 ± 8 vs 30 ± 11 g/beat/m^2^; *P* < 0.001; right ventricular stroke work index [RVSWI]) 5 ± 3 vs 7 ± 4 g/beat/m^2^; *P* = 0.060). Similar trends were observed for thermodilution hemodynamic data on postoperative day 1 with the notable exception of RVSWI, which was no longer predictive of poor outcome (Table 5). Three patients died prior to postoperative day 7, negating the effect their hemodynamics would have had on the data acquired at that time.

**Table 5:** Longitudinal analysis of hemodynamic descriptors*

	3-month outcome		
	non-survivors, mean (SD)	survivors, mean (SD)	*P*
**Preoperative data (prior to skin incision)**			
Cardiac index (L/min/m^2^)	1.9 (0.6)	2.0 (0.7)	0.698
Stroke volume (mL)	51 (18)	48 (19)	0.495
LVSWI (g/beat/m^2^)	17 (7)	20 (9)	0.322
RVSWI (g/beat/m^2^)	6 (3)	6 (3)	0.723
PVR (dyn · s · cm^−5^)	242 (122)	247 (137)	0.814
SVR (dyn · s · cm^−5^)	1287 (470)	1384 (484)	0.479
CVP (mm Hg)	19 (7)	16 (6)	0.190
PCWP (mm Hg)	26 (8)	23 (8)	0.254
**2-hrs post CPB**			
Cardiac index (L/min/m^2^)	3.0 (1.0)	4.0 (1.1)	0.001
Stroke volume (mL)	49 (24)	59 (19)	0.063
LVSWI (g/beat/m^2^)	18 (8)	30 (11)	<0.001
RVSWI (g/beat/m^2^)	5 (3)	7 (4)	0.060
PVR (dyn · s · cm^−5^)	148 (56)	125 (51)	0.162
SVR (dyn · s · cm^−5^)	840 (278)	723 (257)	0.058
CVP (mm Hg)	16 (6)	15 (5)	0.543
PCWP (mm Hg)	17 (5)	17 (5)	0.625
**POD 1**			
Cardiac index (L/min/m^2^)	3.3 (0.9)	3.8 (0.8)	0.075
Stroke volume (mL)	57 (23)	65 (18)	0.132
LVSWI (g/beat/m^2^)	25 (9)	33 (10)	0.007
RVSWI (g/beat/m^2^)	5 (2)	5 (2)	0.702
PVR (dyn · s · cm^−5^)	112 (44)	104 (52)	0.461
SVR (dyn · s · cm^−5^)	779 (213)	830 (299)	0.794
CVP (mm Hg)	15 (4)	15 (4)	0.762
PCWP (mm Hg)	16 (3)	16 (4)	0.606
**POD 7**			
Cardiac index (L/min/m^2^)	3.9 (0.8)	3.4 (0.7)	0.069
Stroke volume (mL)	62 (18)	63 (19)	0.848
LVSWI (g/beat/m^2^)	33 (13)	37 (19)	0.353
RVSWI (g/beat/m^2^)	7 (3)	5 (2)	0.085
PVR (dyn · s · cm^−5^)	121 (34)	119 (44)	0.558
SVR (dyn · s · cm^−5^)	849 (275)	990 (279)	0.100
CVP (mm Hg)	13 (4)	14 (5)	0.439
PCWP (mm Hg)	15 (4)	15 (4)	0.948

*Abbreviations: SD – standard deviation; LVSWI – left ventricular stroke work index; RVSWI – right ventricular stroke work index; POD – postoperative day; PVR – pulmonary vascular resistance; SVR – systemic vascular resistance; CVP – central venous pressure; PCWP – pulmonary capillary wedge pressure; CPB – cardiopulmonary bypass.

### Binary logistic regression

Variables found to be significantly associated with adverse 3-month outcome on univariate analysis were included in a binary logistic regression model. Logistic regression revealed recipient age (*P* = 0.024), serum lactates 2 hours after CPB (*P* = 0.007), and epinephrine use on postoperative day 1 (*P* = 0.007) to be independently associated with 3-month mortality.

## Discussion

Heart transplantation remains the best therapeutic option for patients with end-stage heart failure (4). The scarcity of available donor organs mandates constant refinement of selection algorithms designed to improve long-term results of OHT. Our study aimed to identify pretransplant and perioperative predictors of adverse 3-month outcome in a Croatian academic center. It showed that older recipient age was a significant contributor to poor outcome after OHT, similar to previous publications on the subject (6). We rarely accepted marginal donors in our practice, which may explain why donor age was not predictive of poor outcome. Donor age >50 years has been shown to be a predictor of allograft non-utilization (7). In our study, the proportion of donors aged >50 years was not different between the two cohorts. Similarly, duration of myocardial ischemia was not different between survivors and non-survivors. Croatia is a member of the Eurotransplant framework and organs frequently arrive from other countries. Even with international organ transport, we were able to limit our ischemic times to less than 4 hours in over 80% of transplantations.

In our trial, diabetic patients had marginally inferior outcomes. This point may prove to be even more important in the future, as we are already witnessing the inclusion of increasingly more complex patients into heart transplantation programs (8). Our data on post-transplantation outcome in diabetics support previous publications investigating predictors of mortality (9,10). Our trial also showed the impact of prior sternotomy on worse survival, which is in accordance with earlier data (9). The predictive capacity of inferior creatinine clearance has been illustrated earlier (9). In our study, the impact of worse renal function on mortality was rather close to the level of statistical significance. The impact of recipient and donor sex on postoperative outcomes has been a point of controversy, as has been the importance of sex-specific donor-recipient matching (9,11). Our study did not support any inferior sex-specific outcomes, but was not powered to investigate the potentially important male to female mismatches among donors and recipients.

Serum lactate concentrations are robust markers of hypoperfusion. In our study, lactate levels 2-hours after completion of the procedure proved to be excellent indicators of early post-transplant survival. Maintaining optimal systemic and regional perfusion is of critical importance in the immediate postoperative setting. Our study underscores the fact that even temporary hypoperfusion in the early post-transplantation period may have long-term consequences on patient outcomes. The importance of the early functional performance of the transplanted heart is further corroborated by the increase in 3-month mortality in patients with inferior thermodilution-based perioperative hemodynamic measurements. Optimization of hemodynamics may rely on both pharmacological and mechanical circulatory assistance. The requirement for epinephrine in our practice was an ominous patient characteristic. Epinephrine use in our setting was a surrogate of the severity of underlying hemodynamic compromise, and its association with poor outcome may reflect more on the patient condition than on drug-specific attributes. Analogously, the use of postoperative MCS was limited to patients with the most pronounced reduction of cardiac performance, and hence associated with 3-month mortality. Optimizing MCS timing is pivotal for maintaining good postoperative results. Our study showed that there were patients in whom early institution of mechanical circulatory assistance might be preferable over futile escalation of inotropic support.

Despite the clear shortage of available organs there is continued low utilization of available allografts (7). We were unable to identify any donor-specific criteria that led to inferior outcomes. Currently available evidence supports more liberal use of available allografts, the need for which is accentuated by the increasing number of patients suffering from end-stage heart failure (4).

Our study is a retrospective, single center analysis and therefore is burdened with limitations inherent to this study design. Furthermore, data harvested from institutional databases and medical records relied on the accuracy and comprehensiveness of data input. Some of the examined variables had missing data, which was addressed with multiple imputation methodology. It is possible that some confounding variables were not included in the analysis due to their absence in the available medical records. In addition, our study was not powered to investigate the impact of various sex-matched combinations between donors and recipients (such as male donor/female recipient, female donor/male recipient, sex-matched donor/recipient).

In conclusion, the obtained results after OHT in a tertiary academic center in Croatia showed that recipient age, lower BMI, ischemic cardiomyopathy, reoperation and possibly diabetes and inferior creatinine clearance were pretransplant predictors of 3-month mortality. Epinephrine use, serum lactate concentrations, and re-sternotomy indicated a higher likelihood for an adverse outcome. Conversely, high cardiac output immediately after heart transplantation exerted a favorable effect on 3-month survival. These results highlight the importance of optimizing donor heart performance in the critical post-transplant period.
